# Untoward Long-Term Consequences of Misplaced and Restrictive Annuloplasty for Degenerative Mitral Regurgitation

**DOI:** 10.1016/j.jaccas.2025.103618

**Published:** 2025-06-11

**Authors:** Haruka Sasaki, Hiroyuki Takaoka, Kazufumi Ishida, Shuichiro Takanashi, Yoshio Kobayashi

**Affiliations:** a Department of Cardiovascular Medicine, Chiba University Graduate School of Medicine, Chiba, Japan; b Kawasaki Heart Center, Kawasaki Saiwai Hospital, Kawasaki, Japan

**Keywords:** computer tomography, echocardiography, mitral valve

## Abstract

**Background:**

Mitral stenosis after mitral valve repair for degenerative mitral regurgitation is a poor prognostic factor, but there are few reports of long-term postoperative course.

**Case Summary:**

A 73-year-old man who had undergone mitral valve repair 24 years ago visited our hospital with shortness of breath. Exercise stress echocardiography induced mitral stenosis. Multimodality imaging with computed tomography (CT) and transesophageal echocardiography were really useful in detecting the abnormal location of the prosthetic valve ring causing mitral stenosis.

**Discussion:**

Wide-coverage CT and image processing using a new motion correction algorithm has allowed the visualization of highly mobile structures and thereby enabled more accurate assessment from previous assessments.

**Take-Home Messages:**

The exercise stress test is useful in patients with discrepancies between symptoms, physical examination findings, and the examination results at rest. Morphologic evaluation of valve structures combined with CT and transesophageal echocardiography can provide a more detailed pathophysiologic assessment and understanding of causes.

## Graphical Abstract

**Figure:**
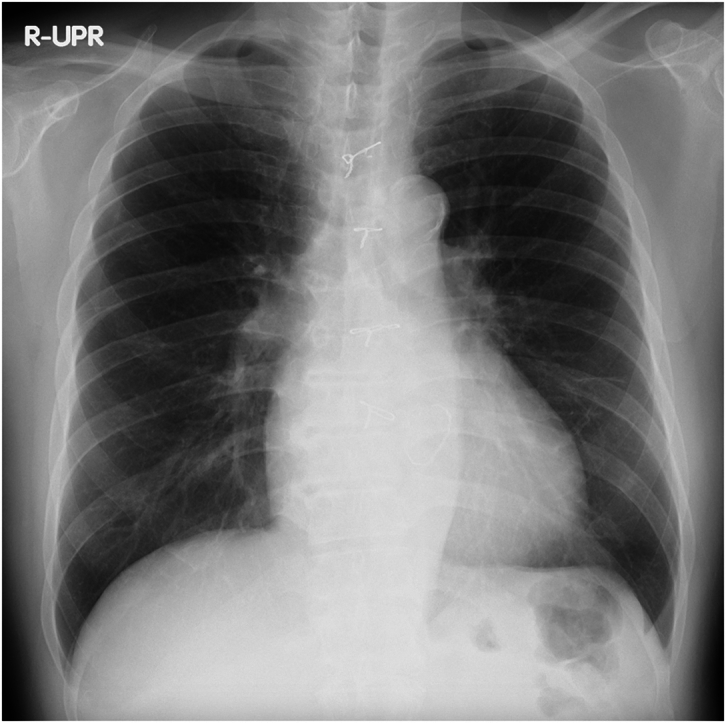

## History of Presentation

A 73-year-old man presented to our hospital complaining of shortness of breath on exertion. The patient had a medical history of severe mitral regurgitation (MR) because of a P3 tendon cord rupture and had undergone mitral valve (MV) repair with resection and suture of the P3 leaflet and annuloplasty using a Duran ring (Medtronic, Minnneapolis, Minnesota) 29-mm prosthetic valve ring at age 49 years. The postoperative course was good, but worsening of tricuspid regurgitation (TR) and enlargement of the right ventricle had been noted on transthoracic echocardiography (TTE) in the most recent several years.

**Take-Home Messages**

• The exercise stress test is useful in patients with discrepancies between symptoms, physical examination findings, and the examination results at rest.
• Morphologic evaluation of valve structures combined with CT and TEE can provide a more detailed pathophysiologic assessment and understanding of causes.

## Medical History

The patient was receiving amlodipine and candesartan for hypertension. Further, he had a history of atrial fibrillation and had undergone catheter ablation twice, at age 61 and 64 years, and was currently receiving anticoagulant therapy.

## Differential Diagnosis

The shortness of breath was considered possibly due to his worsened TR and postoperative MV dysfunction.

## Investigations

**Figure 1:**
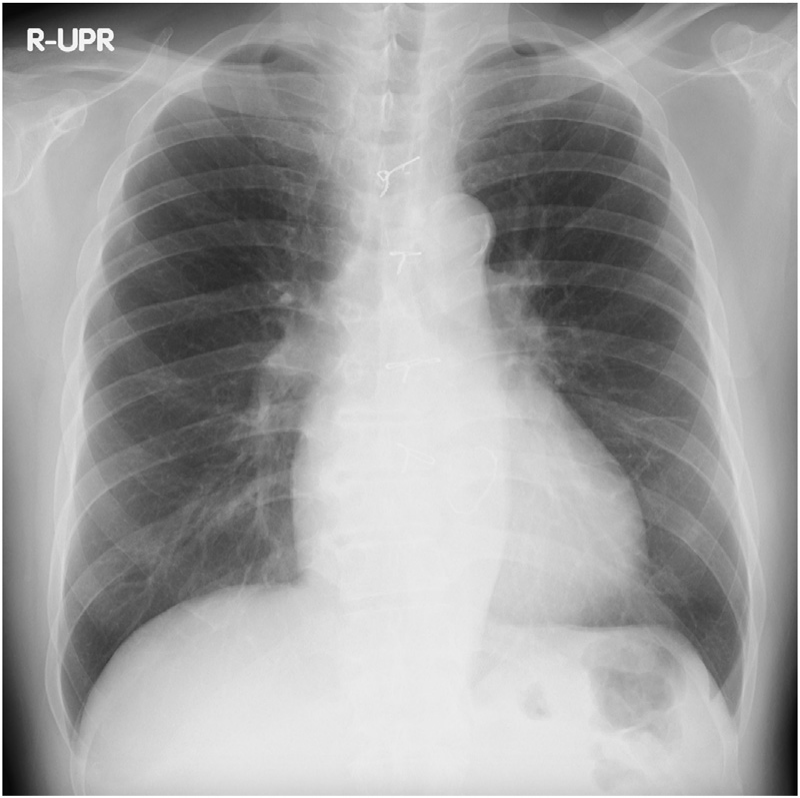
Chest Radiograph on Admission The chest radiograph revealed cardiac enlargement without congestion or pleural effusion.

**Figure 2:**
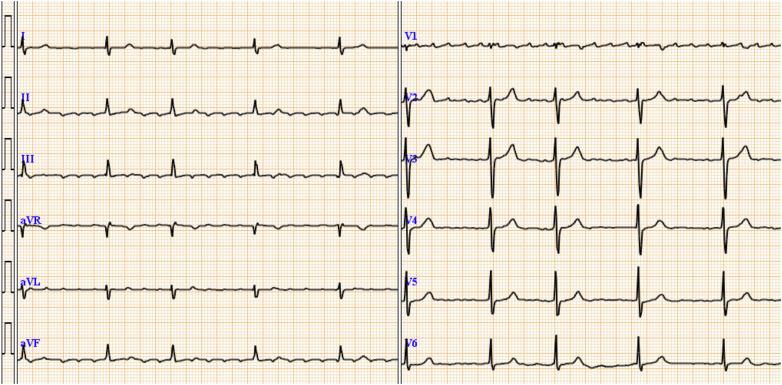
Electrocardiogram on Admission The electrocardiogram showed atrial fibrillation

On physical examination, the patient’s body temperature was 36.5 °C, heart rate was 50 beats/min, and blood pressure was 134/94 mm Hg. A systolic murmur was detected at the right sternal border of the second intercostal space, and there was no evidence of right heart failure symptoms such as jugular venous distention or leg edema. The chest radiograph revealed cardiac enlargement without congestion or pleural effusion (Figure 1), whereas the electrocardiogram showed atrial fibrillation (Figure 2). Laboratory tests revealed a creatinine level of 1.01 mg/dL, blood platelets of 10.5 × 10^4^/μL, hemoglobin of 13.3 g/dL, and B-type natriuretic peptide of 39 pg/mL.

**Figure 3:**
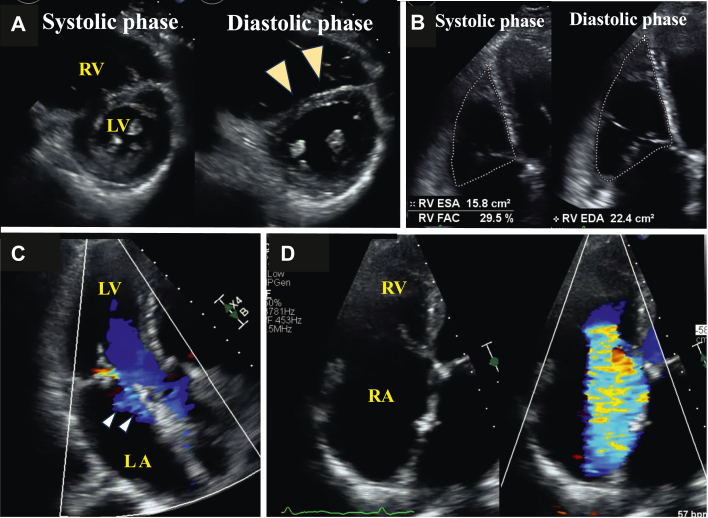
Transthoracic Echocardiography on Admission (A) The ventricular septum was compressed toward the LV side during diastole (arrowheads). (B) RV dysfunction. (C) Mild mitral regurgitation. (D) Torrential tricuspid regurgitation with leaflet tethering. LA = left atrium; LV = left ventricle; RA = right atrium; RV = right ventricle.

**Figure 4:**
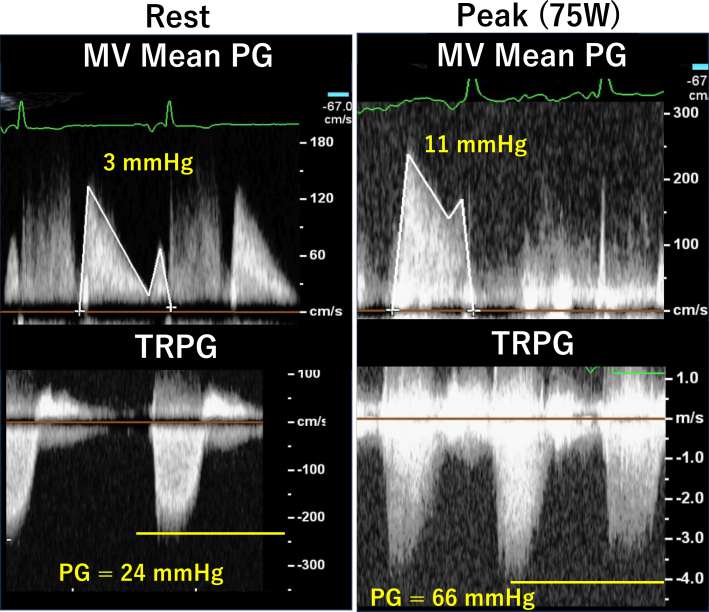
Exercise Stress Echocardiography Findings The MV mean PG was elevated from 3 to 11 mm Hg without worsening of mitral regurgitation. The TRPG was also elevated from 24 mm Hg to 60 mm Hg at a 75-W load on the ergometer. MV = mitral valve; PG = pressure gradient; TRPG = tricuspid regurgitation pressure gradient.

TTE revealed mild left ventricular dysfunction with a left ventricular ejection fraction of 56% and compression of the ventricular septum toward the left ventricle side during diastole, which suggested right ventricular (RV) volume load (Video 1, Figure 3A). The right ventricle was enlarged, and RV function was reduced with an RV fractional area change of 29.5% (Figure 3B). Mild MR (Figure 3C) and mild mitral stenosis (MS) were present, with a mean MV pressure gradient of 3 mm Hg. Both RV enlargement and dysfunction caused tethering of the tricuspid valve leaflets, resulting in loss of coaptation and torrential TR (Figure 3D, Video 2). The inferior vena cava was dilated, but respiratory change was preserved. Exercise stress echocardiography revealed elevation of MV mean pressure gradient to 11 mm Hg and elevation of TR pressure gradient from 24 mm Hg to 66 mm Hg at a 75-W load on the ergometer without worsening of MR (Figure 4).

**Figure 5:**
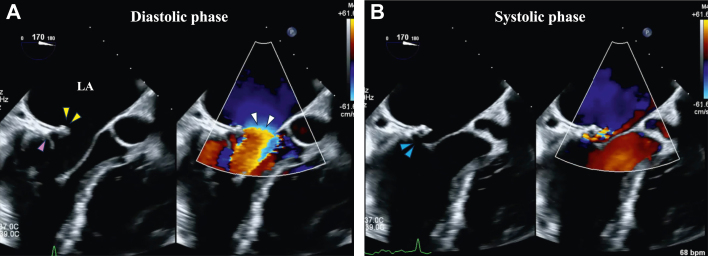
Transesophageal Echocardiography Findings Mid esophageal long axis view in diastolic phase (A) and systolic phase (B). Yellow arrowheads indicate a prosthetic valve ring, pink arrowheads indicate the original mitral valve annulus, white arrowheads indicate accelerated flow, and blue arrowheads indicate acoustic shadow. Abbreviation as in Figure 4.

**Figure 6:**
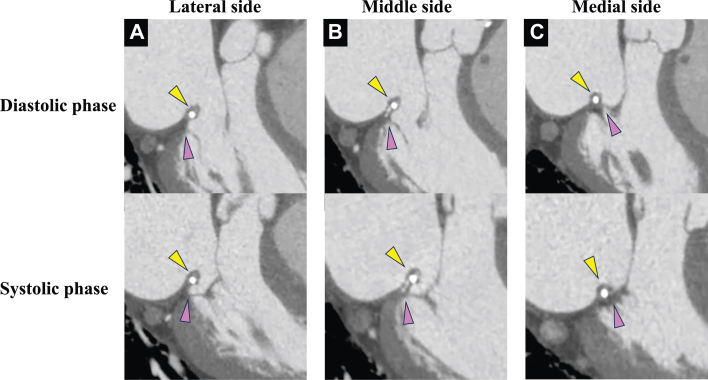
Computed Tomography Findings Lateral side (A), middle side (B) and medial side (C) of mitral valve are shown. Yellow arrowheads indicated prosthetic valve ring and pink arrowheads indicated the original mitral valve annulus.

Transesophageal echocardiography (TEE) revealed the prosthetic valve ring sutured 24 years ago was positioned more to the left atrial side than at the original MV annulus and protruded over the MV (Figure 5, Video 3). Blood flow was accelerated at the level of the protruded prosthetic valve ring. The acoustic shadow of the prosthetic valve ring made it difficult to assess the MV annulus and MV leaflet in some areas. High-resolution cardiac computed tomography (CT) using wide-coverage scanning and a new motion-correction algorithm revealed an abnormal suture location of the prosthetic valve ring in the middle to the lateral side of the MV annulus. In contrast on the medial side, the ring was sutured to the original MV annulus (Figure 6, Video 4).

Based on these results, the cause of his shortness of breath was exercise-induced MS after MV repair. In addition, exercise-induced MS and associated pulmonary hypertension caused RV enlargement and dysfunction, and these resulted in tethering of the tricuspid valve leaflets and loss of coaptation, which was believed to be the cause of his torrential TR. TEE and CT evaluation indicated that the causes of the functional MS were an abnormal sutured position of the previous prosthetic ring in addition to restrictive annuloplasty.

## Management

**Figure 7:**
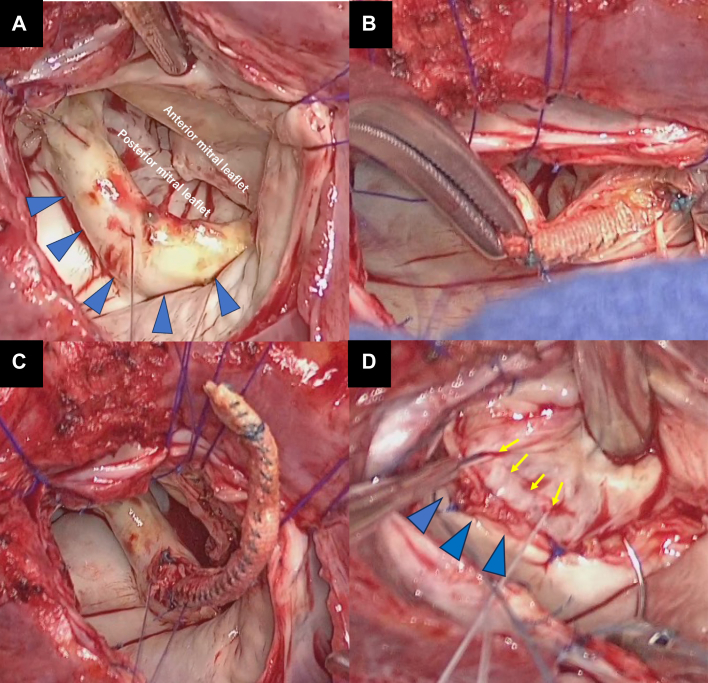
Intraoperative Findings (A) The prosthetic valve ring was covered with thick tissue (blue arrowheads) and (B and C) removed from the inside. (D). The correct valve annulus position (yellow arrows) was different from the previous suture line (blue arrowheads).

Redo MV replacement and TV repair were performed. Intraoperative findings showed that the prosthetic valve ring protruded above the posterior leaflet, which was covered with thick tissue with calcification. The membrane was incised, and the previous prosthetic valve ring was removed from the inside. The surgeon threaded on the correct valve annulus position, which differed from the previous suture line (Figure 7). The posterior mitral leaflet was thickened and had reduced mobility. These findings suggested that the misplaced valve ring had lasted for a chronic course, resulting in long-term stress on the surrounding tissues and MV leaflet.

## Discussion

This is a case of functional MS caused by misplaced and restrictive annuloplasty at the time of MV repair 24 years ago. This caused RV enlargement and dysfunction, resulting in torrential TR. The valve ring abnormality was difficult to detect on postoperative follow-up TTE (Video 1), and other multimodality imaging techniques were used to elucidate the cause.

Exercise stress echocardiography is useful in unmasking functional MS in patients who have undergone MV repair.^1^ Intraoperative findings indicated the abnormal suture position of the previous prosthetic valve ring was the cause of MS. In addition, the 29-mm ring was relatively small compared with the patient's body size, which contributed to the MS. Restrictive annuloplasty should be avoided in degenerative MR because the small prosthetic valve rings cause functional MS, resulting in a poor prognosis.^2^

Investigating the causes of functional MS is an important procedure in decision-making for appropriate treatment. In the present study, CT and TEE were useful in identifying the cause of functional MS. Wide-coverage CT acquires the whole heart image in a single heartbeat and provides good-quality images even in cases with arrhythmia requiring reduced radiation exposure and iodine contrast dose.^3^^,^^4^ The addition of image processing using a new motion-correction algorithm allowed the visualization of highly mobile structures, such as MV leaflets, and thereby enabled a more accurate assessment from previous assessments.^5^ Three-dimensional TEE is a useful tool in assessing MV morphology, but the quality of the image is compromised by the effects of acoustic shadow. In such cases, observation by the transgastric approach could provide better visualization.

## Follow-Up

Postoperative TTE revealed that the prosthetic MV functioned normally and the TR was well controlled by the TV repair. The patient was discharged on postoperative day 8 and periodically followed-up.

## Conclusions

We encountered a male patient with exercise-induced MS because of an abnormal prosthetic valve ring location sutured 24 years previously. A combination of multimodality imaging to investigate the causes of symptoms can provide a more detailed pathophysiologic assessment and understanding of causes.

## Funding Support and Author Disclosures

This work was partially supported by JSPS KAKENHI grant number 22K20498. The authors have reported that they have no relationships relevant to the contents of this article to disclose.

## Appendix

Video 1 Transthoracic echocardiography findings in long axis view The misplaced prosthetic ring could not be detected by transthoracic echocardiography.

Video 2 Transthoracic echocardiography findings in right heart–focused view Right ventricular enlargement and dysfunction caused tethering of the tricuspid valve leaflets, resulting in loss of coaptation and torrential tricuspid regurgitation.

Video 3 Transesophageal echocardiography findings The prosthetic valve ring sutured 24 years previously was positioned more to the left atrial side than the original annulus.

Video 4 Computer tomography findings An abnormal position of the prosthetic valve ring.

## References

[1] Chan K.L., Chen S.Y., Chan V. (2013). Functional significance of elevated mitral gradients after repair for degenerative mitral regurgitation. Circ Cardiovasc Imaging.

[2] Kawamoto N., Fujita T., Fukushima S. (2018). Functional mitral stenosis after mitral valve repair for type II dysfunction: determinants and impacts on long-term outcome. Eur J Cardiothorac Surg.

[3] Annoni A.D., Andreini D., Pontone G. (2018). CT angiography prior to TAVI procedure using third-generation scanner with wide volume coverage: feasibility, renal safety and diagnostic accuracy for coronary tree. Br J Radiol.

[4] Uehara M., Funabashi N., Takaoka H., Komuro I. (2011). Quality of coronary arterial 320-slice computed tomography images compared with 16-slice computed tomography images in subjects with chronic atrial fibrillation. Int J Cardiol.

[5] Suh Y.J., Kim Y.J., Kim J.Y. (2017). A whole-heart motion-correction algorithm: effects on CT image quality and diagnostic accuracy of mechanical valve prosthesis abnormalities. J Cardiovasc Comput Tomogr.

